# Pigeon nidopallium caudolaterale, entopallium, and mesopallium ventrolaterale neural responses during categorisation of Monet and Picasso paintings

**DOI:** 10.1038/s41598-020-72650-y

**Published:** 2020-09-29

**Authors:** Catrona Anderson, Renelyn S. Parra, Hayley Chapman, Alina Steinemer, Blake Porter, Michael Colombo

**Affiliations:** 1 Department of Psychology, University of Otago, P. O. Box 56, Dunedin, New Zealand; 2 Department of Pharmacology and Toxicology, School of Biomedical Sciences, University of Otago, Dunedin, New Zealand; 3 Department of Biopsychology, Institute of Cognitive Neuroscience, Faculty of Psychology, Ruhr-University Bochum, Bochum, Germany

**Keywords:** Cognitive neuroscience, Learning and memory

## Abstract

Pigeons can successfully discriminate between sets of Picasso and Monet paintings. We recorded from three pallial brain areas: the nidopallium caudolaterale (NCL), an analogue of mammalian prefrontal cortex; the entopallium (ENTO), an intermediary visual area similar to primate extrastriate cortex; and the mesopallium ventrolaterale (MVL), a higher-order visual area similar to primate higher-order extrastriate cortex, while pigeons performed an S+/S− Picasso versus Monet discrimination task. In NCL, we found that activity reflected reward-driven categorisation, with a strong left-hemisphere dominance. In ENTO, we found that activity reflected stimulus-driven categorisation, also with a strong left-hemisphere dominance. Finally, in MVL, we found that activity reflected stimulus-driven categorisation, but no hemispheric differences were apparent. We argue that while NCL and ENTO primarily use reward and stimulus information, respectively, to discriminate Picasso and Monet paintings, both areas are also capable of integrating the other type of information during categorisation. We also argue that MVL functions similarly to ENTO in that it uses stimulus information to discriminate paintings, although not in an identical way. The current study adds some preliminary evidence to previous literature which emphasises visual lateralisation during discrimination learning in pigeons.

## Introduction

Categorisation is a useful way of organising and simplifying incoming information from the environment around us^1^. The ability to categorise has previously thought to have been either restricted to humans^2^ or to chimpanzees exposed to language-training^3^. Many other non-human animals, however, have since shown the ability to categorise information. For example, non-human primates can categorise pictures of animals versus non-animals, and food versus non-food objects^1^, as well as complex visual images^4^ and morph images^5,6^. Non-primates such as rats also can categorise arrays of visual stimuli^7^, as well as complex acoustic stimuli^8^. Indeed even bees have the capacity to categorise natural objects such as flowers, plants, and landscapes^9^. The ability to categorize information seems like a fundamental capacity across a range of different animals.

Over the past decade, birds have become a popular non-human animal model for vision due to their highly evolved visual systems and abilities. Some avian brains have been shown to contain around twice as many neurons as mammalian brains of the same size^10^, and avian eyes are large compared to the size of their body^11^, both which lead to the avian brain being highly specialised and efficient for processing visual information. Indeed, birds are able to detect more colours than humans and perceive images at higher resolutions^11^, and can detect subtle luminosity differences that humans cannot^12^.

Herrnstein and Loveland^13^ conducted one of the first studies investigating categorisation in animals in which pigeons were trained to discriminate between photos that contained a human versus photos without a human. Pigeons were not only able to categorise familiar human photos (that is, the ones they were originally trained with), but also generalise this ability to novel human photos. Furthermore, when pictures of humans were pasted into familiar non-human photos, pigeons were still able to categorise these as human photos, indicating that they were discriminating based on whether the photos included humans and not by the fact that certain backgrounds were associated with humans^14^. Beyond discrimination of humans, pigeons have been shown to be able to categorise natural and man-made objects^15^, birds and mammals^16^, and even multidimensional sine wave gratings based on frequency or orientation^17^.

Interestingly, general visual memory, object processing, and visual categorisation in pigeons and other non-human vertebrates have been shown to have a left-hemisphere dominance^18,19^. It seems that the left hemisphere is more involved in identifying local features, uses category-based discrimination, and appears to be where task contingencies are stored, while the right hemisphere relies on configuration and exemplar-based discrimination^18,20^. In birds, these hemispheric asymmetries occur because embryos receive asymmetrical light stimulation during ontogeny, which causes left-dominant asymmetrical projections along the tectofugal pathway to be strengthened^21^.

In what is perhaps one of the classic pigeon categorization studies, Watanabe, Sakamoto, and Wakita^22^ trained pigeons to discriminate between Picasso and Monet paintings using an S+/S− discrimination paradigm. Half of their pigeons were trained to only peck Picasso paintings (Picasso S+), and half were trained to only peck Monet paintings (Monet S+), with each group having as their S− stimuli the paintings from the opposite artist. Watanabe et al.^22^ found that pigeons successfully discriminated between Monet and Picasso paintings even when colour, contour, and sharpness were controlled for. Most strikingly, Watanabe et al.^22^ found that birds maintained their discrimination when presented with novel instances of Picasso and Monet paintings.

In the current study we examined the neural basis of categorization across three different visual areas of the avian brain: the nidopallium caudolaterale (NCL), the entopallium (ENTO), and the mesopallium ventrolaterale (MVL). The NCL is considered to be analogous to primate prefrontal cortex (PFC) on the basis of dopaminergic connectivity and neural architecture^23,24^. Like the PFC, the NCL is implicated in executive functioning^25^ and working memory^26,27^. In primates, PFC activity has been implicated in categorical processing. Freedman et al.^6,28^ trained rhesus monkeys to categorise sets of cat/dog morphed images into the ‘cat’ or ‘dog’ category based on which animal made up the largest proportion of the morphed image. Freedman et al.^6,28^ found that PFC activity differentiated between the cat and dog categories, but also that activity within each category was similar, indicating that the PFC is involved in the categorical representation of information.

A few studies have examined the neural basis of categorization in the avian NCL. Kirsch et al.^29^ trained pigeons on a go/nogo task, in which mandibulation responses to a lightning or heart stimulus (go stimuli) and withholding mandibulation to a triangle or cross stimulus (nogo stimuli) resulted in reward. Kirsch et al.^29^ found that once birds had learned the task, NCL activity discriminated between go/nogo stimuli during their presentation and through the reward period. That is, categorisation was based on stimulus-reward associations. Therefore, we predict that in the current study, NCL will encode categorical information based on similar stimulus-reward associations (or rather, in the case of the current study, category-reward associations).

The ENTO is one of two main intermediary visual areas in the avian brain and is the termination point of the avian tectofugal pathway (analogous to the mammalian colliculothalamocortical pathway^30^). ENTO has been compared to some portion of primate extrastriate cortex, as both ENTO and inferior temporal (IT) cortex in primates have little to no retinotopic mapping and have neurons with large receptive fields^31–33^, and are involved in motion and pattern processing (see Johnston and Colombo^34^ for a review). A number of studies have examined the effects of ENTO lesions on the categorization ability of birds. Watanabe^35^ found that ENTO lesions specifically cause deficits on pseudo-discrimination tasks, but not on food versus non-food discriminations tasks. Watanabe^36^ also found that ENTO lesions cause specific deficits in the ability to discriminate between individual pigeons, but not in the ability to discriminate between pigeons and other bird species. Watanabe^36,37^ argues that ENTO lesions cause a “category-specific agnosia”, especially to categories that are not ecologically important to pigeons, or are new (unlearned) categories. On the basis of these findings, we predict that in the current study, ENTO will encode category-specific information based on visual information, that is, the visual properties of the stimuli within a category.

Finally, MVL is a higher-order visual area that receives projections from ENTO, and has been compared to primate visual extrastriate cortex areas such as V2 and V4, as it is involved in the combined processing of form, colour, and motion^38^. A number of recent studies have examined the role of MVL and other higher-order visual areas in avian cognition. Koenen et al.^39^ recorded neural activity from another higher-order visual area, the nidopallium frontolaterale (NFL), while allowing birds to passively view and categorize stimuli that differed in colour, shape, frequency, and amplitude. Koenen et al.^39^ found that NFL neurons categorically clustered stimuli based on their features. In a more recent study, Azizi et al.^40^ found that neurons in MVL can discriminate between animate and inanimate objects more extensively than ENTO neurons. Furthermore, Azizi et al.^40^ argue that MVL neurons are more sensitive to low-level features, but are also able to distinguish stimuli on a more abstract level than ENTO neurons. Based on these studies, we expect that in the current experiment, MVL will encode categorical information in a similar yet more complex manner than in ENTO. In the current study we will also explore hemispheric differences by recording from both the left and right hemispheres in each of NCL, ENTO, and MVL.

## Method

### Subjects

The subjects were eight experimentally naïve adult homing pigeons (*Columba livia*), and four experimentally sophisticated adult homing pigeons (*Columba livia*). Birds that were experimentally sophisticated had previously served in various studies, including a delayed matching-to-sample task, a serial-order task, and a magnetic study. The birds were housed in individual cages in a colony room. The colony room operated on a 12 h light/dark cycle (lights on at 07:00 and lights off at 19:00), and the temperature was maintained at 20 °C. The birds had access to water and grit at all times, and were fed a mixture of wheat, corn, pellets, grain, and peas to maintain their weight within 80–85% of their free-feeding weight. The experiment was approved by the University of Otago Animal Ethics Committee, and conducted in accordance with the University of Otago’s Code of Ethical Conduct for the Manipulation of Animals.

### Apparatus and stimuli

An operant chamber measuring 350(l) × 430(w) × 390(h) mm internally was used during electrophysiological testing. The stimuli were displayed on a 17-inch monitor at the front of the chamber, which sat behind a Perspex panel. The Perspex panel had six square holes measuring 60 × 60 mm, arranged in a two (row) by three (column) grid. The holes were 65 mm apart, from centre to centre. An infrared touch frame positioned between the Perspex panel and the monitor recorded the XY co-ordinates of all birds’ pecks. Correct responses (i.e. a peck) during S+ trials triggered food delivery via a hopper which was positioned underneath the floor of the chamber, 110 mm below the centre of the bottom-middle square of the Perspex panel. A light positioned next to the hopper was illuminated for 2000 ms while food was delivered.

Stimuli were presented in the top-middle square only, and appeared against the white background of the monitor. There were a total of 14 different stimuli, 7 of which were Monet paintings, and 7 which were Picasso paintings. All of the paintings used were taken from the list of paintings used in the behavioural study by Watanabe et al.^22^. The paintings were in black and white, and had been cropped from one corner so as to best preserve the essence of the original painting. Images were then resized to 100 × 100 pixels.

### Behavioural task

The birds were trained and tested on an S+/S− discrimination task. Half of the birds were trained to peck at Picasso paintings (Picasso S+ group: B2, B10, B5, C8, D10, and D11) and the other half were trained to peck at Monet paintings (Monet S+ group: B9, B11, C3, C4, D12, and D14). Naïve and experienced birds were balanced across the groups. Depending on which S+ group the birds were assigned to, paintings from the other artist served as that birds’ S− stimuli. That is, Picasso S+ birds were also Monet S−, and Monet S+ birds were also Picasso S−.

**Figure 1:**
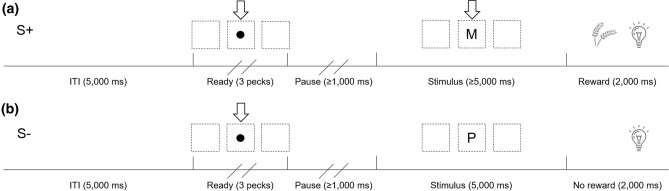
The sequence of events during an S+ trial (**a**) and an S− trial (**b**). Both trials began with a 5000 ms intertrial interval (ITI), followed by the ready period in which a black dot appeared on the screen. After pecking the dot three times, the pause period was initiated in which the birds were required to refrain from pecking for at least 1000 ms. Following the pause period, one of the 14 painting stimuli appeared for a minimum of 5000 ms, during which time pecks were recorded. If an S+ stimulus was displayed, the birds were rewarded following the first peck after 5000 ms, and the stimulus turned off. If an S− stimulus was displayed, they were required to wait 5000 ms until the stimulus disappeared automatically, and were not rewarded. Wheat reward was delivered by a food hopper for 2000 ms on S+ trials, and S− trials had a matching 2000 ms period with no reward. M, Monet painting; P, Picasso painting.

Figure 1 shows the sequence of events on both S+ and S− trials. Trials began with an intertrial interval (ITI) that lasted 5000 ms. At the end of the 5000 ms period, a ‘ready’ stimulus consisting of a small black dot appeared on the screen. This ready stimulus remained on the screen until the bird pecked it three times. After the third peck, the dot disappeared and was followed by a pause period during which the birds were required to refrain from pecking for 1000 ms; pecks before the 1000 ms pause period elapsed reset the timer and continued in that manner until the bird had refrained from pecking for 1000 ms.

Once the pause period ended, one of the 14 stimuli would appear for at least 5000 ms. If the stimulus was the S+, the first peck after 5000 ms would result in 2000 ms access to a wheat reward delivered via an illuminated hopper. If the stimulus was the S−, then the stimulus would automatically disappear after 5000 ms, followed by a 2000 ms period in which the hopper was illuminated but no food delivered. Pecks to the S+ and S− stimuli were recorded during the 5000 ms stimulus period.

Within a session, the seven S+ and seven S− stimuli were randomly presented ten times each, resulting in a total of 140 trials per session. At the completion of each session, behavioural performance was measured using a discrimination ratio (DR), calculated by dividing the number of pecks to all seven S+ stimuli by the total number of pecks to both S+ and S− stimuli.

### Surgery

The birds underwent microdrive surgery after they had reached a DR of at least 0.85 for two consecutive days. Four pigeons had electrodes inserted into the NCL (B2, B9, C3, and C8), four pigeons had electrodes inserted into the ENTO (B5, B10, B11, and C4), and four pigeons had electrodes inserted into the MVL (D10, D11, D12, D14). For each of these regions, two birds were Monet S+ and two were Picasso S+, and two of the birds (one Monet S+ and one Picasso S+) had microdrives installed in the left hemisphere, and the other two birds had microdrives installed in the right hemisphere. For the NCL birds, the electrodes were positioned at AP + 5.5, ML ± 7.5, DV = 1.0. For the ENTO birds, the electrodes were positioned at AP + 9.5, ML ± 6.0, DV = 3.0. For the MVL birds, the electrodes were positioned at AP + 10.5, ML ± 6.0, DV = 0.5.

A mixture of Ketamine (30 mg/kg) and Xylazine (6 mg/kg) injected into the birds’ leg was used as an anaesthetic during surgery. The feathers on the head were then removed to expose the ears and the scalp. A Rezvin stereotaxic adapter^41^ was used to immobilise the head. A topical anaesthetic (10% Xylocaine) was applied to the scalp. The scalp was then cut and retracted to expose the skull. Six stainless steel screws were inserted into the skull, with one of the screws serving as the ground screw. A small hole was drilled into the skull above the target area, and the dura was removed. The electrodes were lowered into the hole until the tips of the electrodes were positioned above the region of interest. The microdrive and screws were covered with dental acrylic before suturing the wound closed. Xylocaine was re-applied, and the pigeons were moved to a heated, padded recovery cage. Once they reached an active state, the birds were returned to their home cages in the colony room where they were allowed to rest for another seven days before recording began.

### Neuronal recording

The microdrives housed eight 25 μm formvar-coated nichrome wires. These wires measured the activity of single neurons. At the beginning of each testing session, we searched for neuronal activity on one of the eight wires, using one of the other wires as the indifferent. Signals were amplified using a Grass P511K amplifier and filtered to remove 50 Hz noise. We recorded from neurons only if their signal-to-noise ratio was at least 2:1. All electrophysiology control, analysis, and storage was accomplished using a Cambridge Electronic Design (CED) system with Spike 2 software. A separate computer was used to control the behavioural task and sent codes to the CED system in order to tag and align the neural data with key task events. The behavioural task during recording was identical to the birds’ training task.

After each session, if a neuron had been isolated and recorded, the electrodes were advanced 40–80 μm before returning the pigeon to their home cage. If no neuron had been isolated, then the electrodes were only advanced 20 μm. Each session usually took around one hour to complete, and the birds completed one session daily, five days a week.

### Histology and electrode track reconstruction

When the electrodes reached the end of the targeted areas, an electrolytic lesion was created by sending a 9 V current through each electrode for 10 s which marked the final recording position. The pigeons were then euthanised using carbon dioxide gas and perfused with a mixture of physiological saline and 10% formalin. Once the brains were removed from the skull, they were kept in 10% formalin for at least 5 days and then sucrose formalin (10% formalin, 30% sucrose) until the brains had sunk. Using a cryostat, the brains were frozen and sliced into 40 µm sections and then stained with Thionin. The position of the electrolytic lesions and depth records were used to reconstruct electrode tracks in each of the birds.

## Results

### Histology

**Figure 2:**
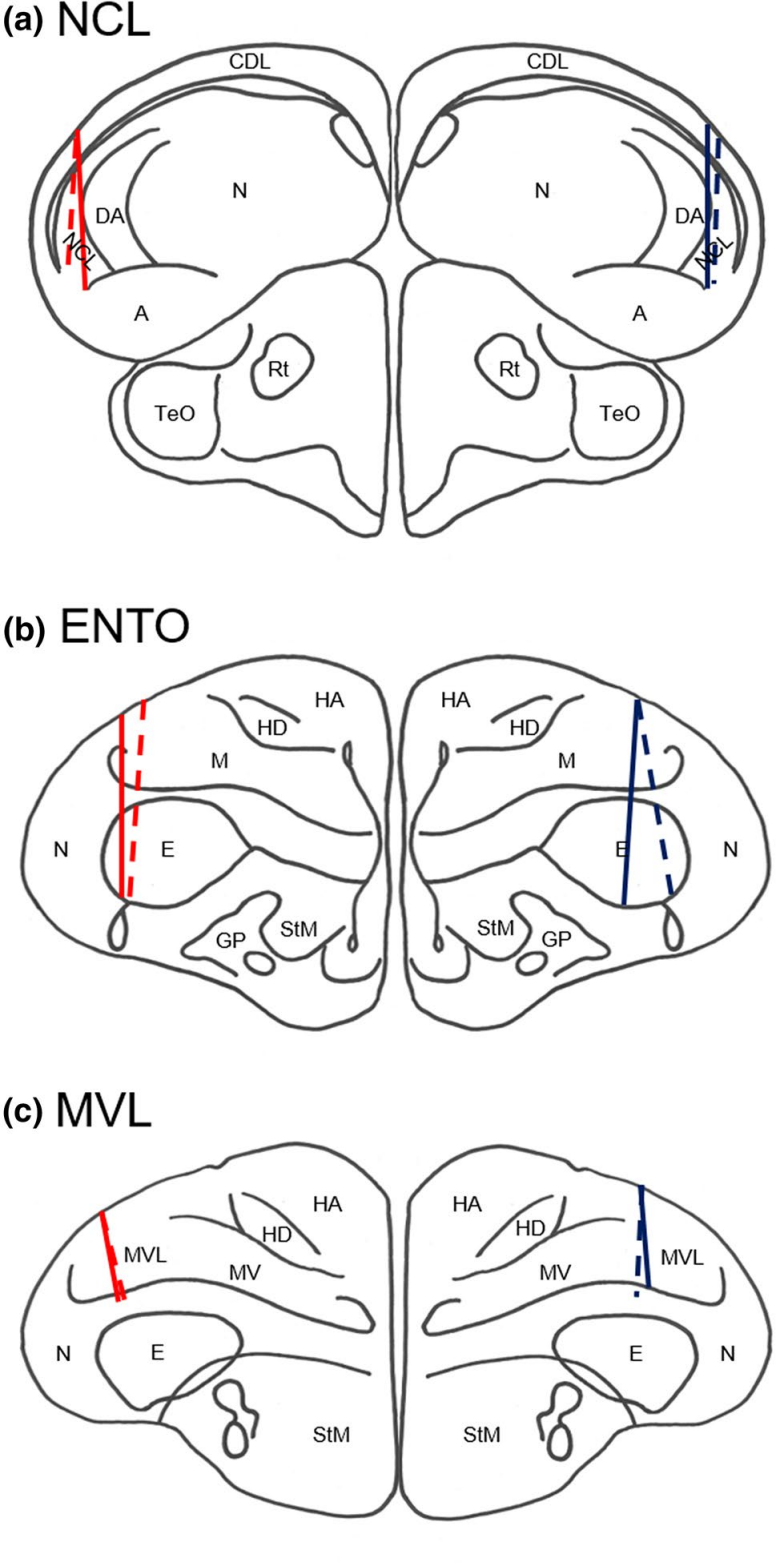
The electrode track reconstruction. (**a**) NCL. Solid red line—C3; dashed red line—B2; solid blue line—B9; dashed blue line—C8. (**b**) ENTO. Solid red line—C4; dashed red line—B10; solid blue line—B11; dashed blue line—B5. (**c**) MVL. Solid red line—D12; dashed red line—D10; solid blue line—D14; dashed blue line—D11. Brain regions (as defined by Reiner et al.^53^): A, arcopallium; CDL, area corticoidea dorsolateralis; DA, tractus dorso-arcopallialis; E, entopallium; HA, hyperpallium apicale; HD, hyperpallium densocellulare; GP, globus pallidus; M, mesopallium; MV, mesopallium ventrale; MVL, mesopallium ventrolaterale; N, nidopallium; NCL, nidopallium caudolaterale; Rt, nucleus rotundus; StM, striatum mediale; TeO, tectum opticum.

All electrode tracks were within the targeted regions as defined by Karten and Hodos^41^. Figure 2 shows the reconstructed tracks for all twelve birds across the three areas.

For NCL, the intended track positions were AP + 5.5 and ML ± 7.5. The track positions for the two left hemisphere birds (B2 and C3) were AP + 6.5, ML + 7.5, and AP + 6.0, ML + 7.5, differing from the intended AP position by 1.0 mm and 0.5 mm, respectively. The track position for one of the right hemisphere birds (B9) was as intended at AP + 5.5, ML − 7.5, while the other bird (C8) was AP + 5.25, ML − 8.0, differing from the intended AP by 0.25 mm and the intended ML by 0.5 mm.

For ENTO, the intended track positions were AP + 9.5 and ML ± 6.0. The track positions for the two left hemisphere birds (B10 and C4) were AP + 9.0, ML + 6.0, differing from the intended AP position by 0.5 mm, and AP + 9.5, ML + 7.0, differing from the intended ML position by 1.0 mm, respectively. The track position for one of the right hemisphere birds (B5) was as intended at AP + 9.5, ML − 6.0, while the other bird (B11) was AP + 9.0, ML − 6.0, differing from the intended AP position by 0.5 mm.

For MVL, the intended track positions were AP + 10.5 and ML ± 6.0. The track positions for the left hemisphere birds (D10 and D12) were AP + 10.5, ML + 7.0, differing from the intended ML position by 1.0 mm, and AP + 10.75, ML + 7.0, differing from the intended AP position by 0.25 mm and the intended ML position by 1.0 mm, respectively. The track positions for the two right hemisphere birds (D11 and D14) were both AP + 10.25, ML − 5.5, differing from the intended AP position by 0.25 mm and the intended ML position by 0.5 mm.

### Behavioural performance

**Figure 3:**
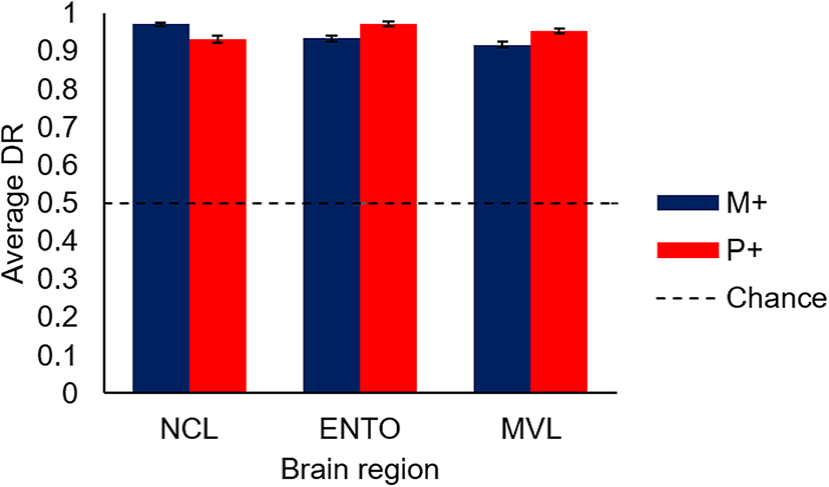
Behavioural performance. Overall behavioural performance on the S+/S− discrimination, as indicated by the discrimination ratio (DR). The dashed line denotes chance performance (0.5), and error bars are ±1 SEM. M+, Monet S+; P+, Picasso S+.

The behavioural performance across all recording sessions of both Monet S+ and Picasso S+ birds, depending on the region they were implanted in, is shown in Fig. 3. All birds discriminated between paintings of the two artists with ease. To see if there was any difference in performance between Monet S+ and Picasso S+ birds, as well as any difference across the birds in the different recorded brain regions, we used a two-way ANOVA with group (Picasso S+ and Monet S+) and region (NCL, ENTO, and MVL) as factors. There was no significant effect of region or group on behavioural performance, nor an interaction effect between the two factors, all *F*s < 1.96, all *p*s > 0.22.

### Neural filtering for visually responsive cells

Neurons with a firing rate of less than 0.2 Hz during the ITI period were removed from the analysis, as well as neurons from incomplete sessions. Neurons were then further filtered based on whether they were visually responsive or not. We conducted two levels of filtering to determine whether neurons were visually responsive. In the first level, we conducted a two-way repeated-measures ANOVA with period (a 300 ms period from the middle of the ITI, and a 300 ms stimulus period) and stimuli (seven Monet and seven Picasso) as factors, with repeated measures over stimuli (Greenhouse–Geisser corrected). The 300 ms stimulus period was taken from + 100 ms after stimulus onset until + 400 ms. We use the start of the stimulus period as opposed to the end as on S− trials we cannot determine whether a bird is still looking at the stimulus at the end of the 5000 ms period. We also start the stimulus period at + 100 ms to avoid the possibility that stimulus information had not yet been processed. If a neuron showed a significant difference in firing rate between the ITI and stimulus period for at least one of the stimuli (i.e. a main effect of period), then the neuron was labelled as being visually responsive.

Although only the first peck to S+ stimuli after 5000 ms results in reward, all birds pecked S+ stimuli during the 5000 ms period. However, the latency to the first peck during the stimulus period varied for each bird, from approximately 300 ms to up to 1000 ms. To better capture the stimulus period for each bird, for the second level of filtering we calculated the minimum average latency to the first peck to S+ stimuli during the 5000 ms period to use for further analysis. On each session that a visually responsive cell was found using the default 300 ms period, the median latency for each of the seven S+ stimuli was calculated, and then these medians were averaged across all S+ stimuli for that session. The minimum of these latencies across all sessions was rounded down to the nearest 50 ms, calculated for each bird separately. For one bird (B5), the latency remained as 300 ms; for two birds (B11 and C8) the new latency was 350 ms; for five birds (B2, B3, B9, D11, and D14) the new latency was 400 ms; for two birds (B10 and D12) the new latency was 450 ms; for one bird (D10) the new latency was 500 ms; and for the final bird (C4) the new latency was 550 ms. We then re-analysed all cells using the same ANOVA above for determining if a cell was visually responsive, but with the new stimulus period lengths for each bird (stimulus periods now varied from + 100 to + 400–650 ms depending on the bird), in case the new stimulus period lengths caused any cells to no longer be classed as visually responsive, or if there were cells that were previously not classed as visually responsive with the original 300 ms period. The cells that were labelled as being visually responsive from this second level of filtering were the neurons used in the final analyses.

### Data analysis

After filtering for visually responsive cells using the new latencies for each bird, a total of 243 neurons were used for data analysis, 71 from NCL, 84 from ENTO, and 88 from MVL. Neurons that displayed significantly greater activity during the sample period compared to baseline ITI activity were labelled as being ‘excitatory’, while neurons that displayed significantly less activity during the sample period were labelled as being ‘inhibitory’. Of the 71 visually responsive NCL neurons, 59 neurons (83.1%) were classed as excitatory, while the remaining 12 neurons (16.9%) were inhibitory. Of the 84 ENTO neurons, 80 (95.2%) were classified as excitatory, and the remaining 4 neurons (4.8%) were inhibitory. Of the 88 MVL neurons, 77 (87.5%) were excitatory and 11 (12.5%) were inhibitory. Due to the extremely low numbers of inhibitory neurons across all three areas, we restricted all subsequent analyses to just the excitatory neurons.

Each trial within a session was divided into 50 ms bins. For each neuron, we then split the sessions’ data into the 14 different trial types (seven Picasso and seven Monet stimuli) and averaged the neural activity within each of these trial types. Thus, average activity to each of the 14 trial types was calculated for each neuron. Data was then normalised by the maximum value in the 5 s ITI within each of the 14 trial types, for each neuron. Because there is no visual stimulation nor any behavioural requirements during the ITI period, we consider ITI activity to represent baseline activity and thus normalised all neural data by the maximum value from this period.

### Population profiles

We constructed population profiles of the excitatory neurons for each bird by averaging across all S+ stimuli and across all S− stimuli for each neuron. To see if there was any difference in neural activity to the Picasso and Monet painting categories, we used a two-way repeated-measures ANOVA with S+S− (S+ stimuli vs S− stimuli) and bin (6 bins for each of the periods; the middle 300 ms of the ITI, the last 300 ms before the first ready peck, the middle 300 ms of the pause period, the last 300 ms of each bird’s specific stimulus period, and the middle 300 ms of the reward period, respectively) as within-subjects factors (Greenhouse–Geisser corrected). We used Keppel’s^42^ modified Bonferroni correction (*p* < 0.02), which was calculated on the basis of ten comparisons (five ANOVAs for each of the five periods: ITI, ready, pause, sample, and reward; for both S+ and S− stimuli). The population profiles are discussed in further detail in the following sections for each region. Note that we do not report results for any main effects of bin, as any significant value just reflects variations in the firing rates from one bin to the next, and is generally not of interest, but we do report interaction effects between S+S− and bin.

**Figure 4:**
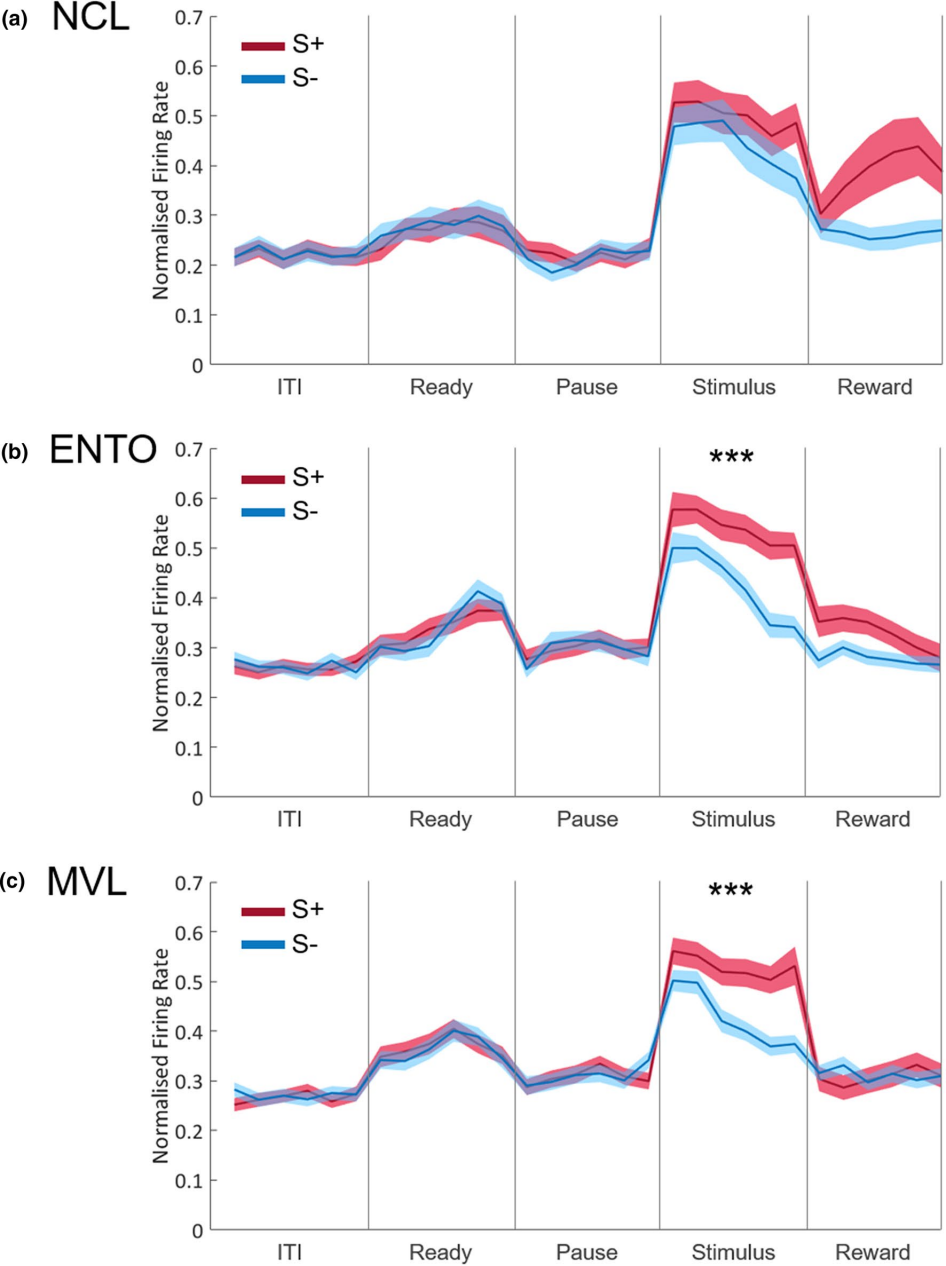
Overall population profiles. The overall population profiles for (**a**) NCL, (**b**) ENTO, and (**c**) MVL. Neuronal data was normalised against the maximum firing rate during the ITI, and bin width is 50 ms. Vertical lines separate the different periods of the task. Shaded bands represent ±1 SEM. ITI, intertrial interval. *p < .02; **p < .01; ***p < .001.

The population profile for NCL is shown in Fig. 4a. There was no significant main effect of S+S− in any of the five periods, all *F*s < 4.90, all *p*s > 0.031. There was a significant interaction effect between S+S− and bin in the reward period, *F*(5,290) = 4.39, *p* = 0.004, with activity on S+ trials increasing across the period, but not on S− trials. There was no interaction effect in the other four periods, all *F*s < 1.54, all *p*s > 0.19.

The population profile for ENTO is shown in Fig. 4b. There was no significant main effect of S+S− in the ITI, ready, pause, or reward periods, all *F*s < 4.73, all *p*s > 0.033. However, there was a significant main effect of S+S− in the stimulus period, *F*(1,79) = 47.94, *p* < 0.001, with activity to S+ stimuli being significantly greater than activity to S− stimuli. Finally, there was a significant interaction effect between S+S− and bin in the stimulus period, *F*(5,395) = 4.43, *p* = 0.001, with activity on S− trials decreasing across the period more rapidly than S+ trials. There was no interaction effect in the other four periods, all *F*s < 2.39, all *p*s > 0.042.

The population profile for MVL is shown in Fig. 4c. There was no significant main effect of S+S− in the ITI, ready, pause, or reward periods, all *F*s < 0.39, all *p*s > 0.53. However, there was a significant main effect of S+S− in the stimulus period, *F*(1,76) = 33.51, *p* < 0.001, with significantly greater activity to S+ stimuli than S− stimuli. Finally, there was a significant interaction effect between S+S− and bin in the stimulus period, *F*(5,380) = 3.29, *p* = 0.016, with activity on S− trials decreasing across the period but not on S+ trials. There was no interaction effect in the other four periods, all *F*s < 2.26, all *p*s > 0.059.

### Hemispheric differences

As a further analysis, we examined whether there were any hemispheric differences in firing patterns within each region. We used a three-way repeated-measures ANOVA with S+S− (S+ stimuli vs S− stimuli) and bin (6 bins for each of the periods; the middle 300 ms of the ITI, the last 300 ms before the first ready peck, the middle 300 ms of the pause period, the last 300 ms of each birds’ specific stimulus period, and the middle 300 ms of the reward period, respectively) as within-subjects factors, and hemisphere (left vs right) as a between-subjects factor (Greenhouse–Geisser corrected). We used the same Keppel’s^42^ modified Bonferroni correction (*p* < 0.02) as in the earlier overall analyses. Again, we do not report results for any main effects of bin, nor any interaction effects between bin and S+S−, as these interactions are identical to those reported from the overall analyses.

#### NCL

**Figure 5:**
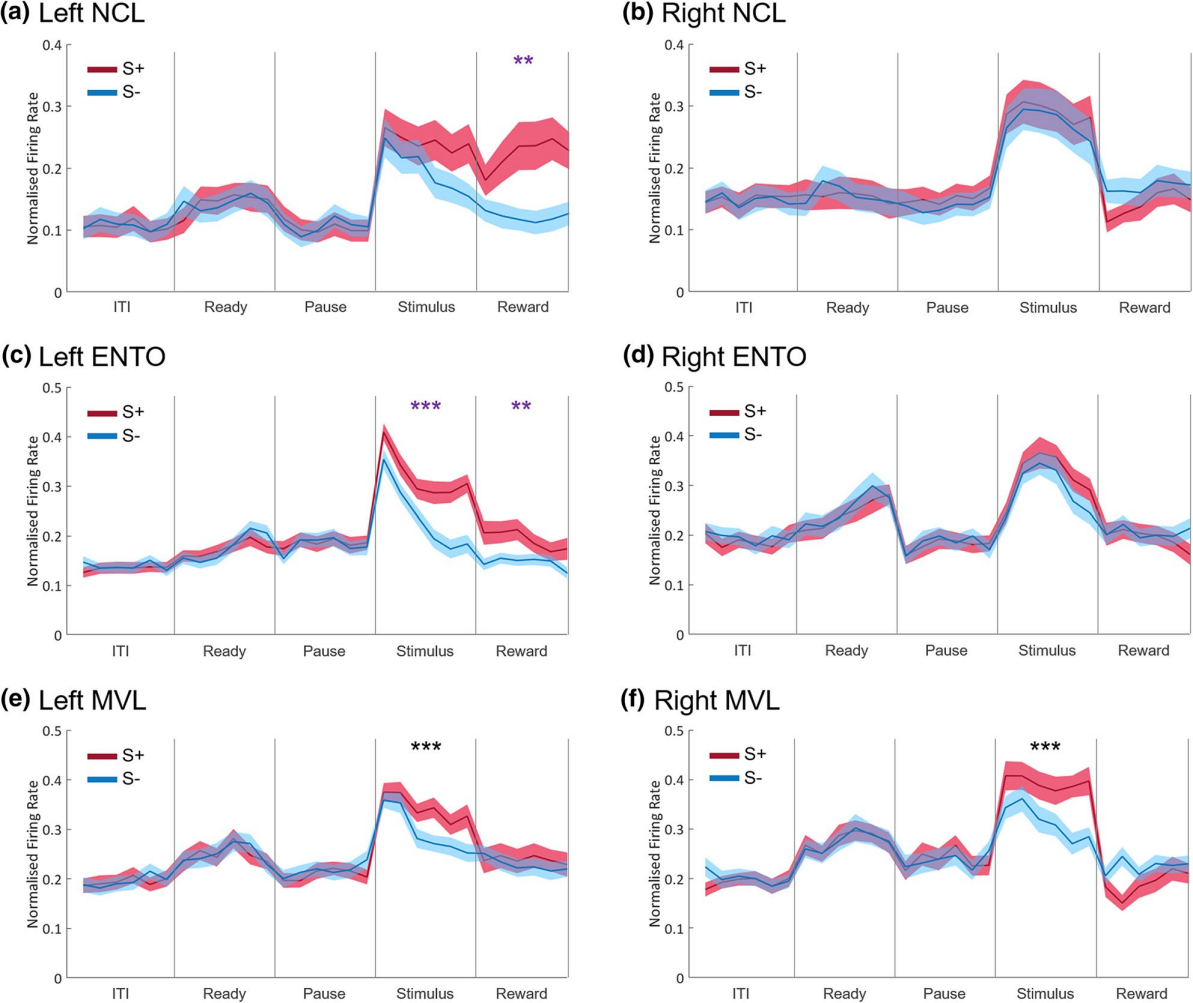
Hemispheric population profiles. The population profiles for left (**a**) and right (**b**) hemisphere NCL, left (**c**) and right (**d**) hemisphere ENTO, and left (**e**) and right (**f**) hemisphere MVL. Neuronal data was normalised against the maximum firing rate during the ITI, and bin width is 50 ms. Vertical lines separate the different periods of the task. Shaded bands represent ± 1 SEM. ITI, intertrial interval. Black asterisks represent significant S+S− differences that are the same across hemispheres, purple asterisks represent significant S+S− differences that are specific to one hemisphere. *p < .02; **p < .01; ***p < .001.

The hemispheric population profiles for left and right NCL are shown in Fig. 5a and b, respectively. There was no significant main effect of S+S− in any of the five periods, all *F*s < 5.24, all *p*s > 0.026, nor a significant main effect of hemisphere in any of the five periods, all *F*s < 3.29, all *p*s > 0.075. However, there was a significant interaction effect between S+S− and hemisphere in the reward period, *F*(1,57) = 10.67, *p* = 0.002, but not in any of the other four periods, all *F*s < 4.29, all *p*s > 0.043. To further understand the interaction effect found in the reward period, we used paired *t*-tests to see whether there was any difference between S+ and S− trials in each hemisphere. In the left hemisphere, there was a significant difference between S+ and S− trials, with greater activity to S+ over S− trials, *t*(28) = 3.22, *p* = 0.003. In the right hemisphere, there was no difference between S+ and S− trials, *t*(29) = 1.13, *p* = 0.27. Finally, there was no interaction effect between bin, S+S−, and hemisphere in any of the five periods, all *F*s < 2.88, all *p*s > 0.021.

#### ENTO

The hemispheric population profiles for left and right ENTO are shown in Fig. 5c and d, respectively. There was a significant main effect of S+S− in the stimulus period, *F*(1,78) = 49.84, *p* < 0.001, but not in any of the other four periods, all *F*s < 2.59, all *p*s > 0.11. There was a significant main effect of hemisphere in the ITI, *F*(1,78) = 10.16, *p* = 0.002, and ready period, *F*(1,78) = 9.99, *p* = 0.002, but not in the other three periods, all *F*s < 2.35, all *p*s > 0.13. Finally, there was a significant interaction effect between S+S− and hemisphere in the stimulus period, *F*(1,78) = 12.35, *p* = 0.001, and the reward period, *F*(1,78) = 5.77, *p* = 0.019, but not in the other three periods, all *F*s < 0.65, all *p*s > 0.42. To further understand the interaction effect found in the stimulus and reward periods, we used paired *t*-tests to see whether there was any difference between S+ and S− trials in each hemisphere. In the left hemisphere, there was a significant difference between S+ and S− trials in the stimulus period, *t*(38) = 9.52, *p* < 0.001, and in the reward period, *t*(38) = 2.98, *p* = 0.005, with greater activity to S+ over S− trials in both periods. In the right hemisphere, there was no significant difference between S+ and S− trials during either period, both *t*s < 2.16, both *p*s > 0.037. Finally, there was no interaction effect between bin, S+S−, and hemisphere in any of the five periods, all *F*s < 1.97, all *p*s > 0.091.

#### MVL

The hemispheric population profiles for left and right MVL are shown in Fig. 5e and f, respectively. There was a significant main effect of S+S− in the stimulus period, *F*(1,75) = 44.87, *p* < 0.001, but not in any of the other five periods, all *F*s < 0.69, all *p*s > 0.41. There was no main effect of hemisphere in any of the five periods, all *F*s < 2.00, all *p*s > 0.16. There was also no significant interaction effect between S+S− and hemisphere in any of the five periods, all *F*s < 3.05, all *p*s > 0.085. Finally, there was no interaction effect between bin, S+S−, and hemisphere in any of the five periods, all *F*s < 2.19, all *p*s > 0.067.

## Discussion

We analysed a total of 243 visually responsive neurons, 71 from NCL, 84 from ENTO, and 88 from MVL. In each of these areas, the majority of neurons were excitatory (83.1%, 95.2%, and 87.5% of neurons in NCL, ENTO, and MVL, respectively). These excitatory neurons were further analysed using a two-way repeated-measures ANOVA (Greenhouse–Geisser corrected) to see whether neural activity differed between S+ and S− stimuli within each task period. In NCL, we found that overall activity to S+ and S− stimuli did not differ within any period. In ENTO and MVL, we found that overall activity to S+ and S− stimuli only differed within the stimulus period, in which activity to S+ stimuli was greater than activity to S− stimuli.

We predicted that the NCL would encode categorical information based on behavioural outcomes such as reward. Interestingly, we found that the overall population of NCL neurons did not distinguish between Picasso and Monet paintings in any period. However, when hemisphere was added as a factor to the analyses, we found differences in activity to S+ and S− stimuli during the reward period. Left hemisphere neurons showed greater activity during S+ trials than during S− trials, but right hemisphere neurons fired equally to S+ and S− trials. Like with visual memory, pigeons as well as in chickens^43^, quails^44^ and zebra finches^45^, have been shown to have a left-hemisphere dominance during simple reward-related discriminations^46,47^. In fact, the left hemisphere appears to be specialised for discriminating important learned stimuli (such as food) from distractor stimuli (such as pebbles or grit), while the right hemisphere is more easily distracted by novel stimuli^18,48^. Note that for half the birds, the S+ stimuli were Picasso paintings and for the other half they were Monet paintings, indicating that the increased activity towards S+ stimuli was not solely based on stimulus properties (e.g. all Picasso paintings are more interesting than all Monet paintings), but also on which paintings were part of the rewarded category (S+ stimuli vs S− stimuli). We have previously shown that reward-based coding is more dominant in NCL, and that when there is the opportunity to code information in this way (due to differential reward outcomes), NCL neurons more or less default to reward coding over stimulus coding^26,27^. Therefore, as in Kirsch et al.^29^, NCL neurons are likely categorising information based on stimulus-reward associations, rather than the visual properties of the stimuli in each category.

We predicted that ENTO would encode categorical information based on category-specific information, that is, visual differences between Monet and Picasso paintings. We found that the overall population of ENTO neurons only distinguished between the two categories of paintings in the stimulus period, with neural activity being significantly more excitatory towards S+ stimuli than S− stimuli. The difference in activity in the stimulus period suggests that ENTO can distinguish between the two categories of paintings, likely based on differences in the visual properties of the paintings. The idea that ENTO is an area which processes stimulus information over reward information is consistent with the findings of many other studies^26,35,36^. Furthermore, we also found a significant difference between S+ and S− trials in both the stimulus and reward periods when hemisphere was added as a factor to the analyses. In both the stimulus and reward periods, left hemisphere neurons showed greater activity during S+ than S− trials, while right hemisphere neurons fired equally to S+ and S− trials. A left-hemisphere dominance for visual categorisation in birds has been well-established^18–20^, but ENTO has also been shown to have a stronger left hemisphere dominance in a colour discrimination task when differential reward outcomes were strengthened^49^. Reward processing in a visual area is not unprecedented; while visual areas such as ENTO may primarily process visual/stimulus information, both ENTO and other visual areas such as the Wulst have been shown to be modulated by reward information^26,50^. Due to the nature of the S+/S− discrimination task, reward is intrinsically tied to stimulus information, causing both types of information to be useful in distinguishing between the two categories. Therefore, ENTO neurons appear to encode categorical information based on category-specific information, but that this category-specific information is not limited to visual information.

Finally, we predicted that MVL would encode categorical information in a similar yet more complex manner than in ENTO. Like in ENTO, we found that the overall population of MVL neurons only distinguished between the two categories of paintings in the stimulus period, with neural activity being significantly more excitatory towards S+ stimuli than S− stimuli. Similarly, we argue that MVL also encodes categorical information based on category-specific information. Based on overall population profiles, there is little that would distinguish the coding properties of MVL from the coding properties of ENTO. Interestingly, MVL was the only area that did not display a left-hemisphere dominance; both hemispheres displayed a significant difference in activity during the stimulus period, with greater activity to S+ stimuli over S− stimuli. Also, unlike ENTO, MVL did not display any differences in activity during the reward period. Perhaps the fact that significant categorical processing only occurs in the stimulus period for both hemispheres indicates a stronger ability for MVL to categorise based on visual properties than in ENTO. MVL has been shown to be sensitive to visual features of stimuli and intrinsically categorise information based on those features^39,40^. On the other hand, ENTO has been shown to be specifically involved in forming new categories^35–37^, which in an S+/S− discrimination task would be greatly facilitated by incorporating reward information. ENTO lesions also cause deficits in pseudo-categorisation tasks^35^, in which categorisation would rely heavily on identifying individual stimuli rather than viewing all stimuli in one category as the same. It is possible that higher-order visual areas like MVL only use stimulus information to categorise objects, compared to ENTO which perhaps incorporates some reward information as well. While we do not directly compare the three areas to one another, our results indicate that all three areas appear to be involved in similar yet distinct steps of the categorisation process. ENTO has reciprocal projections with both NCL^51^ and MVL^52^, and therefore it is likely that reward-related information about the categories is shared between NCL and ENTO, and stimulus-related information about the categories is shared between MVL and ENTO.

It should be noted that while our results indicate a strong left hemisphere dominance for categorisation in both NCL and ENTO, these results should be considered as being preliminary in nature. Due to the small number of subjects, the number of neurons from each hemisphere are relatively small and may not represent the population as a whole. The small number of neurons in each hemisphere may be why we have found no hemispheric differences in MVL, and with larger number of neurons, hemispheric differences may emerge. Furthermore, due to the limits of single-unit electrophysiology, we are unable to compare neurons from both hemispheres within individual subjects, which may mean that there is a degree of individual variability within these results. However, the fact that we find such strong differences in NCL and ENTO when directly comparing hemispheres should not be discounted, especially considering previous literature which clearly shows that categorisation is lateralised to the left hemisphere in birds.

In conclusion, our results indicate that ENTO and MVL neurons primarily use stimulus information to discriminate between Monet and Picasso paintings, while NCL neurons are likely using reward information to drive this discrimination. However, ENTO is also able to incorporate reward information to help discriminate two categories. On a hemispheric level, we found preliminary evidence that ENTO and NCL show a strong left-hemisphere dominance, in that left hemisphere neurons categorise Monet and Picasso paintings more strongly than right hemisphere neurons. In MVL, we did not find any hemispheric asymmetries in categorising paintings, which may reflect more complex visual categorisation occurring in higher-order visual areas. Overall, it is apparent that both visual and working memory areas of the pigeon brain are involved in the categorisation of Picasso and Monet paintings.
